# ATTITUDES TOWARD AGING, RESILIENCE, AND SELF-RATED HEALTH OF CHINESE OLDER ADULTS: FINDINGS FROM HONOLULU AND WUHAN

**DOI:** 10.1093/geroni/igz038.2189

**Published:** 2019-11-08

**Authors:** Keqing Zhang, Wei Zhang, Sizhe Liu, Bei Wu

**Affiliations:** 1 University of Hawaii at Manoa, Honolulu, Hawaii, United States; 2 New York University, New York, New York, United States

## Abstract

A growing body of literature found that attitudes towards aging is related to individual’s health among older adults. This paper aims to examine the relationship between negative attitudes towards aging and self-rated health among Chinese older adults living in Honolulu (N=400) and Wuhan (N=552) and explore the moderating roles of resilience and gender. Results showed that the positive relationship between attitudes towards aging and self-rated health was moderated by resilience such that higher levels of resilience weakened this association significantly and substantially, implying its stress-buffering role. Both the positive focal relationship and the moderating effect appeared to be stronger among older adults in Honolulu than their peers in Wuhan. For gender differences, the moderating effect of resilience was only significant for males in Honolulu and females in Wuhan, indicating that males in Honolulu and females in Wuhan may rely heavily on psychological resources such as resilience in coping with stressors.

